# A Normalized Spot-Sample Estimation of α-1 Antitrypsin Clearance: The Search for a Simpler Test in Protein Losing Enteropathy

**DOI:** 10.1055/s-0045-1813661

**Published:** 2025-11-23

**Authors:** Anjilivelil J. Joseph, Anoop John, Junita Rachel John, Julie Hephzibah, Ebby George Simon, Amit K. Dutta, Sudipta Dhar Chowdhury, Reuben Thomas Kurien, Dilip Abraham

**Affiliations:** 1 Department of Gastroenterology, Christian Medical College and Hospital, Vellore, Tamil Nadu, India; 2 Department of Nuclear Medicine, Christian Medical College and Hospital, Vellore, Tamil Nadu, India; 3 Department of Wellcome Trust Research Laboratory, Christian Medical College and Hospital, Vellore, Tamil Nadu, India

**Keywords:** protein losing enteropathy, antitrypsin, elastase, scintigraphy, hypoalbuminemia

## Abstract

**Introduction:**

Protein losing enteropathy (PLE) is usually a diagnosis of exclusion, which requires cumbersome tests to confirm. In the quest for a simpler diagnostic test, we hypothesized that a spot stool sample estimation of α-1 antitrypsin will be sufficient to make a diagnosis of PLE, if we control for serum α-1 antitrypsin concentration and degree of stool dilution.

**Materials and Methods:**

Consecutive patients with a clinical suspicion of PLE and who had been advised a scintigraphy study were recruited after getting informed consent. The study excluded patients less than 1 year of age, pregnant women, and those with a clinical suspicion of chronic pancreatitis. Serum α-1 antitrypsin, spot stool α-1 antitrypsin, and stool elastase was assessed in all the patients. The diagnostic value of the index test was estimated from the patients with positive scintigraphy scan compared with a negative scan, expressed as sensitivity and specificity and the area under the receiver operating characteristic curve (AUROC).

**Result:**

A total of 33 patients underwent scintigraphy with a clinical suspicion of PLE. Twenty patients (60%) showed tracer activity in the gut suggestive of PLE. Spot stool α-1 antitrypsin below 0.26 mg/g had a sensitivity of 100% to rule out PLE; however, the specificity was only 46%. Spot stool α-1 antitrypsin/(serum α-1 antitrypsin * elastase) ratio performed similar to spot stool α-1 antitrypsin as a diagnostic test (AUROC: 0.814 [0.61–1.0] vs. 0.796 [0.54–1.0]).

**Conclusion:**

Random stool antitrypsin is a sensitive test for diagnosing PLE; however, it lacks specificity. Spot stool α-1 antitrypsin/(serum α-1 antitrypsin * stool elastase) does not provide any additional value in the diagnosis of this syndrome.

## Introduction

Protein losing enteropathy (PLE) is a syndrome where there is excessive loss of protein from the gastrointestinal (GI) tract. It can be caused by various disorders and include erosive and nonerosive GI disease and those due to lymphatic obstruction. 1 In a patient with hypoalbuminemia, PLE is usually a diagnosis of exclusion once proteinuria, decreased protein synthesis (i.e., liver cirrhosis), and malnutrition are ruled out. Confirmation of PLE is cumbersome and is established by a 24-hour α-1 antitrypsin clearance and/or a radionuclide scintigraphy using Technetium-99m ( ^99m^ Tc)-labeled human serum albumin (HSA). Protein loss scintigraphy is the more commonly used test and is considered the gold standard in diagnosis of PLE.

α-1 antitrypsin is neither secreted nor absorbed in the bowel, and its presence in stool is an indicator of PLE. It is not degraded by digestive enzymes but can be degraded by acid in hypersecretory states. Estimation of faecal loss of α-1 antitrypsin uses a 24-hour clearance method to account for variability in serum concentration of α-1 antitrypsin and differences in stool consistency. In the quest for a simpler diagnostic test, we hypothesized that a spot stool sample estimation of α-1 antitrypsin will be sufficient to make a diagnosis of PLE if we control for serum α-1 antitrypsin concentration and degree of stool dilution. As an estimate of stool dilution, we used stool elastase concentration, which does not get affected by mucosal disease or lymphatic hypertension.

## Materials and Methods

This was a prospective study conducted by the Department of Gastroenterology and Nuclear Medicine between 2016 and 2022. Consecutive patients with a clinical suspicion of PLE and who had been advised a scintigraphy study were recruited after getting informed consent. The study excluded patients less than 1 year of age, pregnant women, and those with a clinical suspicion of chronic pancreatitis. Details regarding demographics, clinical profile, and laboratory and radiologic parameters were noted onto standard proformas. The patient gave one stool and one serum sample on the morning of the scintigraphy scan. The study was approved by the institutional review board, IRB number 10253 dated 05.09.2016.

### Technetium-99m-Labeled Human Serum Albumin Scintigraphy

Freshly prepared ^99m^ Tc-labeled HSA was given intravenous (740 MBq), followed by a saline flush. Prior to injection of radiotracer, quality control using thin layer chromatography method was performed and a radiochemical purity above 95% was ensured. Images were acquired in gamma camera (GE Optima 640) with low-energy high-resolution collimator, with a matrix of 256 × 256. For cases with difficulty assessing bowel activity in static images, single-photon emission computerized tomography scans were obtained. Static images were acquired in anterior view at 10, 30, 90 minutes (early phase) and 24 hours (delayed phase). Anterior images of the neck were also acquired at 10 minutes to rule out presence of free technetium (which could be assessed on the basis of uptake in the thyroid gland).

Images were evaluated with respect to visualization and initial appearance time of abnormal radioactivity. If tracer activity was noted in the gut, the scan was considered positive. If doubtful tracer accumulation was noted in the scintigraphy then it was considered as suspicious for PLE.

### Spot Stool α-1 Antitrypsin/(Serum α-1 Antitrypsin * Stool Elastase Ratio)

Commercial enzyme-linked immunosorbent assay (ELISA) assays were used for estimating levels of α-1 antitrypsin in stool. Stool aliquots for the faecal biomarker ELISA assays were supplemented with a cocktail of protease inhibitors before being stored at −70°C. Stool α-1 antitrypsin levels in diluted faecal samples were estimated using a quantitative sandwich ELISA (ImmuChrom, GmbH) as per manufacturer's instructions. Stool α-1antitrypsin levels were expressed in units of milligram/gram (mg/g) of faeces.

Commercial ELISA assays were used for estimating levels of stool elastase. Stool elastase levels in diluted faecal samples were estimated using a quantitative sandwich ELISA. Stool elastase levels were expressed in units of microgram/gram (µg/g) of faeces.

The patient was asked to take 3 days of tablet lansoprazole 30 mg once daily and 50 g butter for 3 days prior to the day of the protein scintigraphy scan and collection of stool sample.

### Statistical Analysis

Data entry was done using Microsoft Excel. The data were analyzed in SPSS (Version 25: IBM Corp., Armonk, New York, United States). Descriptive statistics for categorical variables were reported using frequency and percentage. Continuous variables that are normally distributed were reported using mean ± standard deviation. Non-normal variables were reported using median (interquartile range). Categorical variables were compared using chi-square test, and continuous variables were compared using Mann–Whitney tests. The diagnostic value of the index test was estimated from the patients with positive scintigraphy scan compared with a negative scan, expressed as sensitivity and specificity and the area under the receiver operating characteristic curve. A *p* -value of <0.05 was regarded as statistically significant.

## Results

**Fig. 1:**
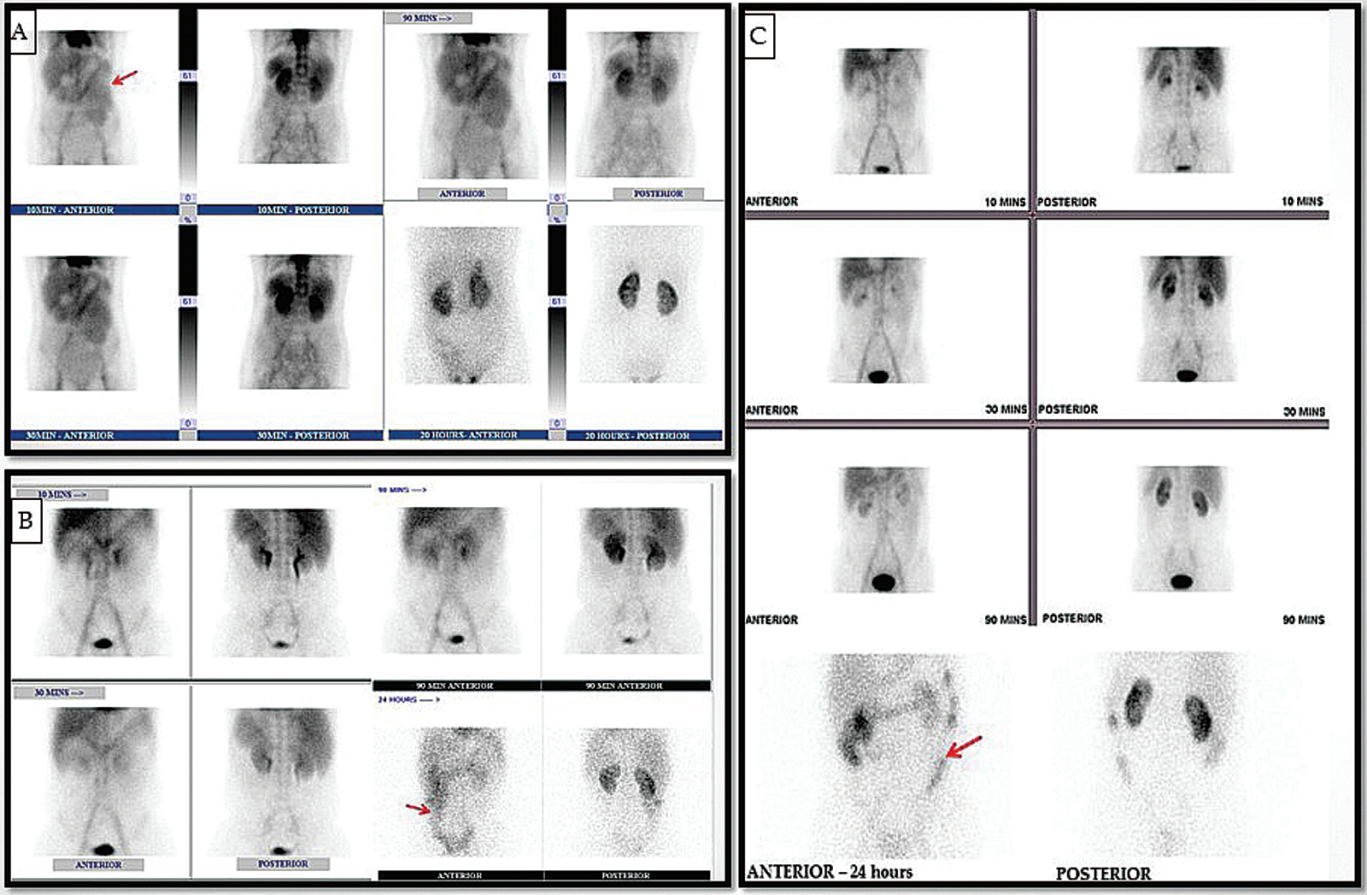
Anterior scintigrams demonstrate: ( **A** ) A 48-year-old lady with anasarca, loss of weight, and appetite—suspected PLE. Initial images at 10 minutes showed protein leak in the small bowel loops (jejunum). ( **B** ) A 34-year-old gentleman with hypoalbuminemia—initial images showed no abnormal tracer activity; however, 24-hour imaging showed abnormal tracer activity in the large bowel—the patient was found to have an underlying etiology of abdominal tuberculosis. ( **C** ) A 60-year-old gentleman with ascites ruled out liver disease and renal protein loss; 24-hour imaging showed abnormal tracer activity in the large bowel—suggestive of protein loss through the bowel loops. PLE, protein losing enteropathy.

During the study period, 33 patients underwent scintigraphy with a clinical suspicion of PLE. Twenty patients (60%) showed tracer activity in the gut suggestive of PLE ( Fig. 1 ). Tables 1 and 2 show the baseline characteristics and laboratory findings of these patients, respectively.

**Table 1:** Baseline characteristics

	Scintigraphy positive	Scintigraphy negative
Number of patients	20	13
Age, years a	34 (range: 3–69)	45 (range: 16–69)
Male/female	13/7	4/9
Chief presenting symptoms		
Diarrhea	9 b c	9 ^µ^ f
Abdominal pain	2 d	1 g
Loss of weight	1	0
Anemia	2	0
Peripheral edema	4 b	5 e
Ascites	5 c	1 f
Peripheral edema and ascites	4 d	2 g
Fever	0	1
Biochemical hypoalbuminemia	1	0
Duration of symptoms, years a	1 (range: 0.1–12)	0.5 (range: 0.1–5)
Etiology of PLE		
Crohn's disease	5	
Intestinal lymphangiectasia	3	
Abdominal tuberculosis	2	
Systemic lupus erythematosus	1	
Celiac disease	1	
Congestive enteropathy	1	
Cronkhite Canada syndrome	1	
Incomplete evaluation	6	

Abbreviation: PLE, protein losing enteropathy.

a Median.

b Two positive patients presented with both diarrhea and peripheral edema.

c Five positive patients presented with both diarrhea and ascites.

d One positive patient presented with abdominal pain, edema and ascites.

e Four negative patients presented with both diarrhea and peripheral edema.

f One negative patient presented with both diarrhea and ascites.

g One negative patient presented with abdominal pain, edema and ascites.

**Table 2:** Laboratory findings

	Scintigraphy positive ( *n* = 20)	Scintigraphy negative ( *n* = 13)	*p* -Value
Total white blood cell count (cells/mm ^3^ ) a	8,335 (2934)	8,715 (3077)	0.72
Neutrophil/lymphocyte ratio a	7.5 (11.1)	4.0 (2.5)	0.27
Protein (g/dL) a	4.0 (0.96)	4.9 (1.3)	0.03
Albumin (g/dL) a	1.8 (0.61)	2.0 (0.8)	0.51
Albumin/globulin ratio a	0.91 (0.39)	0.79 (0.48)	0.44
Prealbumin (mg/dL) ( *n* = 19) a	7.4 (6.8)	5.9 (3.8)	0.48
Prealbumin/albumin ratio ( *n* = 19) a	4.1 (3.4)	3.4 (2.8)	0.53
Serum α-1 antitrypsin (mg/dL) ( *n* = 27) b	106.5 (12–199)	103 (12–184)	0.72
Spot stool α-1 antitrypsin (mg/g) ( *n* = 33) b	3.7 (0.3–48.1)	0.3 (0.07–20.4)	0.004
Spot stool α-1 antitrypsin/serum α-1 antitrypsin ratio ( *n* = 27) b	5.6 (0.2–35.3)	0.27 (0.1–11.1)	0.013
Spot stool α-1 antitrypsin/elastase ratio ( *n* = 33) b	32.5 (3.3–1840)	4.6 (0.2–121)	0.006
Spot stool α-1 antitrypsin/(serum α-1 antitrypsin elastase) ratio ( *n* = 27) b	33.3 (4.5–1360)	6.5 (0.2–65.9)	0.013

a Mean (standard deviation).

b Median (range).

**Fig. 2:**
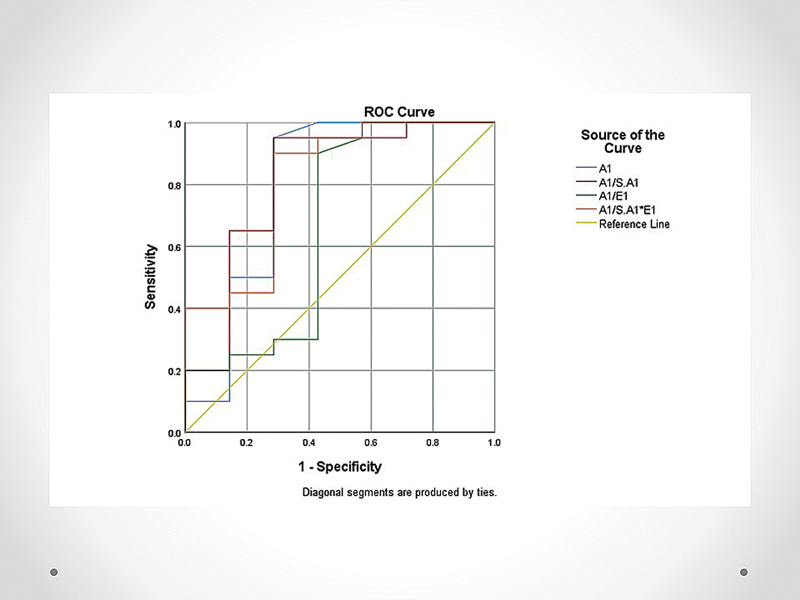
ROC curve comparing diagnostic tests. ROC, receiver operating characteristic.

Spot stool α-1 antitrypsin below 0.26 mg/g had a sensitivity of 100% to rule out PLE; however, the specificity was only 46%. Table 3 and Fig. 2 shows the comparison of the diagnostic tests.

**Table 3:** Diagnostic tests area under the receiver operating characteristic curve

	Observations a	ROC area (CI)	Cutoff	Sensitivity (%)	Specificity (%)
Spot stool α-1 antitrypsin (mg/g)	27	0.796 (0.54–1.0)	≥0.587	90	72
Spot stool α-1 antitrypsin/serum α-1 antitrypsin ratio	27	0.814 (0.59–1.0)	≥0.82	90	72
Spot stool α-1 antitrypsin/elastase ratio	27	0.668 (0.38–0.95)	≥6.3	90	58
Spot stool α-1 antitrypsin/(serum α-1 antitrypsin * elastase) ratio	27	0.814 (0.61–1.0)	≥8.6	90	72

Abbreviations: CI, confidence interval; ROC, receiver operating characteristic.

a Only 27 patients had serum α-1 antitrypsin levels.

## Discussion

PLE poses a diagnostic as well as a therapeutic challenge for the gastroenterologist. The presentation is varied and is usually related to the underlying etiology. The diagnosis of PLE is commonly based on the detection of protein loss into the gut using albumin that is tagged to a radioactive tracer. These scans can also help identify the site of protein loss.

Alpha-1 antitrypsin is a protease inhibitor, which is resistant to degradation by gut proteases. Studies have shown that the estimation of fecal α-1 antitrypsin concentration can be used as a screening test for PLE. 2 The stool α-1 antitrypsin concentration is influenced by stool consistency, serum α-1 antitrypsin level, and intestinal permeability. Estimation done on a random specimen of stool does not accurately estimate fractional α-1 antitrypsin excretion. This was shown in a study by Strygler et al, where stool α-1 antitrypsin concentration did not correlate with the α-1 antitrypsin clearance in 21% of the patients. 3 A more accurate estimate of GI protein loss is the α-1 antitrypsin clearance rate, but its use is limited by the need for a cumbersome and impractical 72-hour stool collection. By correcting for stool dilution and serum concentrations, we postulated that a new derived parameter would accurately reflect GI protein loss. Although the reasoning seemed robust, in actual fact, the new model did not perform better than a random α-1 antitrypsin concentration in our patients.

α-1 antitrypsin is inactivated at a pH less than 3 and so cannot be used as a test to diagnose protein losing gastropathy. Hence, we used lansoprazole to raise the intragastric pH and maintain the activity of gastric α-1 antitrypsin as in an earlier study. 4 However, in our study, we did not have any patients with gastric protein loss. All patients in the study underwent testing after a fatty meal. Protein loss due to intestinal lymphangiectasia occur because of the rupture of intestinal lymphatics. This is more pronounced after a fatty meal, which increases the enteric lymph flow, which in turn causes elevated lymphatic pressure and leakage of lymph into the bowel lumen. 5

Prealbumin/albumin ratio has been shown to be significantly higher in patients with PLE, though in our study, it was not statistically different between the groups. 6 Hence, our ordeal in the diagnosis of PLE continues and we have to rely on a positive scintigraphy scan for the confirmation of PLE in a patient. ^99m^ Tc-labeled HSA scintigraphy is an important diagnostic modality for diagnosis of PLE since its introduction in 1986. 7 This method is simple and sensitive not only to diagnose PLE, but also to localize the site of protein loss in the GI tract, which is almost impossible in certain cases with other imaging modalities. 8 Due to the intermittent nature of protein loss from the gut in PLE, it is important to include both early and delayed phases in the study. 9 The pooled sensitivity of scintigraphy was been found to be 100% and hence used as the gold standard in our study. 10 11

Since our institution is a referral center for nuclear medicine imaging, some patients attended our hospital only for the GI protein loss scintigraphy test and returned to their respective hospitals for further follow up. So, we were not aware of the etiological diagnosis in a small proportion of patients. Additionally, fecal protein loss severity was not correlated with the scintigraphy. Few studies have assessed the severity of protein loss based on the intensity of uptake (using liver and kidney uptake as reference). However, a recent study showed that the intensity of abnormal tracer activity does not always correlate with disease severity as explained by the fact that protein-losing enteropathy is characterized by nonselective depletion of all plasma proteins, and the disease severity is affected by turnover rates of various types of proteins, such as albumin and immunoglobulin. ^99m^ Tc albumin scintigraphy visualizes only the depletion of albumin and not all plasma proteins, thereby not making it the ideal tracer for quantification of severity. 12 These were the limitations of our study. Various other diagnostic tools have been utilized to identify a possible source of protein loss in PLE. Double-balloon enteroscopy has been shown to have a higher diagnostic yield to determine the etiology of PLE and capsule endoscopy has been shown to be useful as a screening test and for follow-up of PLE without any stricture. 13 14 15 Recently, more advanced endoscopy such as confocal laser endomicroscopy has improved the diagnostic yield by helping to diagnose diseases without obvious mucosal lesions and having compromised epithelial barrier. 16 Hence, ^99m^ Tc-labeled HSA scintigraphy continues to be the most reliable test in the diagnosis of PLE and the benchmark against which further tests would have to be compared.

## Conclusion

PLE poses a diagnostic challenge to the gastroenterologists, since it involves many cumbersome tests. Random stool antitrypsin is a sensitive test for diagnosing PLE; however, it lacks specificity. Spot stool α-1 antitrypsin/(serum α-1 antitrypsin * stool elastase) does not provide any additional value in the diagnosis of this syndrome. A simple diagnostic test for PLE continues to evade us.
